# Fractionation of Extracellular Polymeric Substances by Aqueous Three-Phase Partitioning Systems

**DOI:** 10.1021/acs.iecr.4c00840

**Published:** 2024-06-06

**Authors:** Evelyn C. Antunes, Bruna Cintra, Matthieu Bredel, Hardy Temmink, Boelo Schuur

**Affiliations:** † Wetsus—European Centre of Excellence for Sustainable Water Technology, Oostergoweg 9, 8911MA Leeuwarden, The Netherlands; ‡ Department of Environmental Technology, Wageningen University and Research, Bornse Weilanden 9, 6708 Wageningen, The Netherlands; § Sustainable Process Technology Group, Department of Chemical Engineering, Faculty of Science and Technology, University of Twente, Drienerlolaan 5, 7522 Enschede, The Netherlands

## Abstract

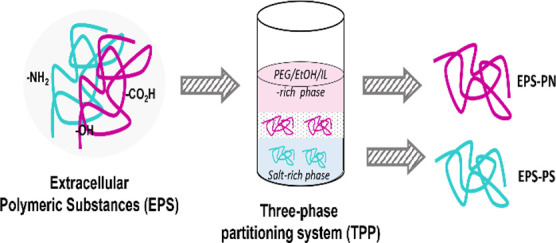

Extracellular polymeric substances (EPS) are natural polymers secreted by microorganisms and represent a key chemical for the development of a range of circular economy applications. The production of EPS comes with notable challenges such as downstream processing. In this work, a three-phase partitioning (TPP) system was investigated as a fractionation technique for the separation of polysaccharides and proteins, both present in the EPS culture broth. The effect of the type of phase-forming compounds (alcohol, polymer, or ionic liquid, in combination with salt) and its concentration were evaluated and compared to the results previously obtained with model systems. The recyclability of phase-forming compounds used to form the fractionation platform was assessed by ultrafiltration. The best fractionation of EPS was achieved using a TPP system composed of 23 wt % ethanol and 25% K_3_C_6_H_5_O_7_ as 82% EPS-PS partitioned to the salt-rich/bottom phase, and 76% EPS-PN was recovered as an interfacial precipitate, which could be readily resolubilized in water. This represented an increase of 1.24 and 2.83-fold in the purity of EPS-PS and EPS-PN, respectively, in relation to the initial feed concentration. Finally, high recovery yields of phase-forming compounds (>99%) and fractionated EPS (>80%) were obtained using ultrafiltration/diafiltration (UF/DF) as the regeneration technique. The substantial fractionation yields, selectivity, and recyclability of the phase-forming compounds confirm the potential of TPP systems in combination with UF/DF as the separation method for real biopolymer mixtures. Key contributions of this study include the demonstration of the applicability of a readily scalable and cost-effective separation technique for the fractionation of EPS from real EPS-containing broths, while also the limitations of prescreening with model systems became clear through the observed deviating trends between model system studies and real broth studies.

## 1 Introduction

Biopolymers, also known as natural polymers, are macromolecules formed by linking either one or a combination of a variety of repeating units (e.g., amino acids or hydroxy fatty acids). They can be extracted from a large variety of sources, such as animals, plants, or microorganisms.^1^ Promoting the use of biopolymers aligns with the UN’s Sustainable Development Goals (SDGs)^2^ as they possess desirable features in relation to the SDGs, namely, they are obtained from renewable source(s), can theoretically be produced with a reduced carbon footprint as compared to fossil-based polymers (requiring all production aspects to be carbon-lean) and, in some cases, are biodegradable^3^

In particular, the production of natural polymers by microorganisms has gained great attention among researchers due to a series of advantages over their plant- and animal-based counterparts.^1^ Some advantages are that their production is less climate- or seasonal-dependent, and they present a smaller footprint and a higher growth rate.^1,4^ Natural polymers produced by microorganisms also have a higher structural diversity, which may unravel even more applications.^1,5,6^ Microbial biopolymers can be found intracellularly (e.g., polyphosphate and polyhydroxyalkanoates^7^) or secreted by the cells as in the case of many polysaccharides, proteins, and glycoproteins.^8,9^ The latter type of biopolymers is known as extracellular polymeric substances (EPS).^10^

EPS can be produced by pure cultures^11−13^ or mixed microbial cultures (MMCs).^14−17^ Pure cultures are the most common approach to obtain high-quality commercial biopolymers, such as alginate, dextran, and xanthan.^18^ However, growing pure cultures is significantly more expensive because they require sterilization conditions and utilization of expensive carbon substrates. On the other hand, MMCs are based on the principle of natural selection and, thus, do not require sterile conditions.^19^ This implies that less complex and less energy-demanding growth processes may be applied, resulting in lower process costs. Another advantage of employing MMCs relies on their ability of using diverse carbon sources, which allows valorization of waste streams as substrates, which generates additional potential for economic circularity and lowers the overall process cost.^3,19^

EPS produced by MMCs currently have potential for various applications, such as a metal adsorbent for mining and metallurgy industries,^20^ a flocculating agent in wastewater treatment plants,^17,21,22^ and a carbon source for the biotechnological production of other valuable chemicals.^9^ To fully unlock the commercial potential and expand the range of applications of EPS produced by MMCs, the downstream processing, a key production step which is still costly,^23−25^ needs to be further developed.

Downstream processing of EPS generally starts by separating biomass from a culture medium supernatant (by filtration or centrifugation), followed by extraction and fractionation steps.^23−25^ The extraction step aims at separating biopolymers from the original matrix and can involve both physical (sonification and heating) and chemical methods (alkaline treatment using NaOH and formamide).^26,27^ Finally, the fractionation step aims at separating different types of biopolymers (for instance, separating polysaccharides from proteins). To achieve this, several separation techniques are available, such as membrane processes,^28,29^ precipitation,^30,31^ chromatography,^32,33^ and liquid–liquid extraction,^34−36^ depending on the targeted product.

Most of the reported fractionation approaches for EPS are not suitable for large-scale application as they have been developed for analysis of the biopolymers. Such fractionation methods are often based on the precipitation of proteins by trichloroacetic acid or chloroform, followed by precipitation of polysaccharides by alcohol or acetone.^11,12,23,37,38^ The use of chlorinated hydrocarbons as solvents in large amounts as compared to the amount of EPS is certainly not sustainable, and there is a need for better separation methods. For instance, Kumar et al.^39^ recovered acidic polysaccharides from the EPS matrix by ethanol precipitation. No quantitative assessment regarding the efficiency of the method was reported. Du et al.^40^ obtained EPS’s polysaccharides with purity of 99.6% by employing a fractionation approach also based on trichloroacetic acid and ethanol precipitation, followed by dialysis. Bahl et al.^31^ showed that exopolysaccharides could be precipitated by isopropanol with a yield of 81% and at a solvent ratio of 9:1 (v/v). In these studies, valorization of only one class of biopolymers was achieved, as the analysis of these biopolymers was the target of these studies. Because the scope of most of the studies in the field is to develop fractionation protocols for analytical purposes, rather than designing a scalable cost-effective separation process,^23^ there is a lack of knowledge regarding scalable and sustainable approaches for the fractionation of EPS in production processes.

Aqueous two-phase systems (ATPS) represent a potential and scalable technique for the fractionation of EPS.^41,42^ ATPS consist of a type of liquid–liquid extraction highly suitable for biomolecules since both coexisting phases are mostly composed of water.^43−45^ The biphasic system is formed by mixing two water-soluble compounds that are incompatible together in solution above a certain concentration.^43−45^ ATPS can be formed by combining a variety of compounds, such as ionic liquid, polymer, and alcohol, along with (in)organic salts.^43,44^ The fractionation of EPS using ATPS offers several advantages over the conventional fractionation methods developed for analytical purposes, such as biocompatibility, use of greener chemicals, and easier scaling-up.^45^ The effectiveness of ATPS for the fractionation of biopolymer, from a variety of sources, has been confirmed by studies using ionic liquid-,^34,41,46^ polymer-,^35,47^ and alcohol-^48,49^ based ATPS. For instance, Kee et al.^50^ used a polymer-salt ATPS (PEG 1000/(NH_4_)_2_SO_4_) to recover keratinase (microbial exoenzyme) from its culture broth, and 78% of protein partitioned to the PEG-rich phase. Wu et al.^51^ reported simultaneous separation of polysaccharides and proteins from the fermentation broth by ionic liquid-salt ATPS (([Ch][Cl])/K_2_HPO_4_). The authors observed that about 69% of polysaccharide migrated to the salt-rich phase, while about 82% of protein was found in the IL-rich phase. In addition, a comparison between ATPS and conventional fractionation approach (based on precipitation using ethanol and chloroform) was also carried and found that ATPS led to a product with higher purity.^51^

ATPS can also be designed in such a way that one of the targeted molecules is retrieved as the interfacial precipitate, as happens in three-phase partitioning (TPP) systems.^52−55^ TPP systems combine salting-out phenomena and solvent precipitation to separate and concentrate biopolymers at the interface. Even though the ATPS/TPP combined approach seems highly promising, it has received little attention by researchers. A few studies have reported this technique, as done by Suarez Ruiz et al.^54^ in their investigation about the fractionation of microalgal biopolymers by polymer/ionic liquid ATPS/TPP. The authors showed that 94% of proteins were found as the interfacial precipitate in the PPG400/[Ch][DHP] system. Belchior and Freire^55^ also studied the potential of the ATPS/TPP approach for the fractionation of biopolymers (more specifically egg white proteins). The system was composed of PEG 2000 and phosphate salt and led to an 82% recovery yield of ovalbumin in the PEG-rich phase and 77% of lysozyme as a precipitate at the interface.

To the best of our knowledge, the use of the ATPS/TPP approach as a fractionation technique for EPS produced by MMCs has not yet been explored beyond our own studies with model compounds,^41,42^ i.e., no studies with real EPS-rich broths have been reported. It is anticipated that by fractionating the different classes of biopolymers present in the EPS matrix, full valorization can be achieved, and other applications (potentially with more added-value) may be unlocked beyond the current applications for unfractionated EPS mixtures. For the development of ATPS/TPP, next to the fractionation itself, the isolation of the targeted biopolymers and recycling of chemicals required during the fractionation step are also to be investigated. Even though these aspects are essential for the process feasibility, they are typically not included in studies in the field.^56^

This work describes the use of the ATPS/TPP approach for the fractionation of real (EPS) produced in an aerobic bioreactor into a polysaccharide-rich fraction and a protein-rich fraction. In our previous publications,^41,42^ the ATPS/TPP approach was used to fractionate model biopolymer mixtures (i.e., mixture of bovine serum albumin and alginate or dextran), and it led to promising results. In this work, a validation and application study was carried out, aiming at systematically confirming whether and how the various systems selected in the studies based on the model component behave in the presence of real EPS obtained from mixed microbial broths, in face of their full compositional complexity. Another key aspect that has been investigated is whether the phase-forming compounds can be regenerated properly in the presence of real EPS. The recycling of phase-forming compounds was carried out by ultrafiltration/diafiltration. This study provides new important insights into how to proceed from model compound studies to scalable separation techniques for EPS produced in the aerobic bioreactor, which can contribute to designing cost-effective fractionation techniques for EPS produced by mixed microbial cultures.

## 2 Materials and Methods

### 2.1 Chemicals

TPP systems were based on three types of ATPS: alcohol-, ionic liquid-, and polymer-based ATPS. The alcohols used as the phase-forming compound were ethanol (C_2_H_6_O, purity ≥99.8%, VWR) and 2-propanol (C_3_H_8_O, purity ≥98%, VWR). The ionic liquids used were 1-butyl-3-methylimidazolium acetate ([C_4_mim][CH_3_CO_2_] purity = 98%, IoLiTec), 1-butyl-3-methylimidazolium bromide ([C_4_mim]Br, purity = 99%, Alfa Aesar), and 1-butyl-3-methylimidazolium chloride ([C_4_mim]Cl, purity = 98%, Acros Organics). The polymers used were poly(ethylene glycol) 400 g/mol (PEG400, purity = n/a, Sigma-Aldrich) and poly(ethylene glycol) 1000 g/mol (PEG1000, purity = n/a, Sigma-Aldrich). (In)organic salts were also employed to ensure the formation of a biphasic system. The salts used were ammonium sulfate ((NH_4_)_2_SO_4_, purity = n/a, VWR), dipotassium phosphate (K_2_HPO_4_, purity = n/a, VWR), tripotassium citrate (K_3_C_6_H_5_O_7_, purity ≥99%, Alfa Aesar), and tripotassium phosphate (K_3_PO_4_, Sigma-Aldrich).

Samples of EPS were obtained from another project within our research group, in which the production of EPS from synthetic wastewater was studied, and from which the results have been reported elsewhere.^17^ In summary, after cultivation, EPS were separated from the culture broth by centrifugation and extracted using cation exchange resin. After that, the samples were dialyzed and freeze-dried to obtain dry solids. Before fractionation, in this work, EPS was resolubilized in milli-q water by overnight stirring. The composition of the obtained EPS aqueous mixture is shown in Table 1.

**Table 1:** Composition of Extracellular Polymeric Substance Aqueous Mixture Prior Fractionation Experiments

total dissolved organic carbon (g L^–1^)	1.01
biopolymer (%w/w)	97.10
low molecular weight molecules (%w/w)	2.76
humic acid (%w/w)	0.05
total polysaccharide (g glucose equiv L^–1^)	1.13
total protein (g BSA equiv L^–1^)	0.30

### 2.2 EPS Fractionation Experiments

The selection of ATPS with the potential to form a TPP system was done based on the data of ternary phase diagrams available in the literature,^57−60^ and their use for the fractionation of model mixtures containing only BSA and alginate has been proven.^41,42^ Minimizing the use of phase-forming compounds was also considered for the selection of the mixture point. The results from our previous study using model components were also considered as leading, as the concentrations at which two phases formed were expected to be similar. Prior to the experiments, an EPS solution was obtained by resolubilizing the freeze-dried EPS in milli-q water. The preparation of each partitioning system was carried out by weighing an appropriate amount of the phase-forming solutes and EPS solution in 15 mL tubes, mixing thoroughly using a vortex mixer, and then leaving equilibrating for 60 min. This time has been reported as sufficient for achieving equilibrium in ATPS.^52,61−64^ Following equilibration, complete phase separation was achieved by centrifugation (4500 rpm for 10 min), and the phases were carefully separated using a syringe. The concentration of polysaccharides and proteins in both bottom and top phases was determined by the analytical methods described in detail in Section 2.5. The concentration of EPS in the interface precipitate was obtained by a mass balance. All experiments were carried out at room temperature (21 ± 1 °C). The experiments were performed in duplicate, and the results were reported as the average of two independent assays with their respective standard deviation.

### 2.3 Regeneration Experiments

Diafiltration using an ultrafiltration membrane was employed to separate the fractionated EPS from the phase-forming compounds. Dead-end filtration was carried out using a polysulfone membrane in a stirred cell module (10 mL) at 3 bar and room temperature (21 ± 1 °C). A membrane of 100 kDa molecular weight cutoff (MWCO) (Mycrodin Nadir) was used to filter the salt-rich phase. The dimensionless diafiltration volume (DV) was defined as the total volume of water added to the cell during filtration divided by the initial feed volume. The permeate flux could not be determined due to practical constraints (i.e., equipment limitations).

### 2.4 Analytical Methods

A Pierce BCA Protein Assay Kit (Thermo Fischer) was used to quantify the protein concentration in the samples. The absorbance of the mixture was measured at 562 nm using a microplate spectrophotometer (Victor3 1420 Multilabel Counter, PerkinElmer). The polysaccharide concentration was determined by phenol-sulfuric acid assay,^65^ using glucose as the standard for the calibration curve. The absorbance of the mixture was measured at 490 nm by using a microplate spectrophotometer. Interferences caused by the phase-forming compounds were handled by filtering samples prior to analysis, using Amicon ultra centrifugal filters (MWCO 10 kDa, Merck Millipore).

### 2.5 Calculation Methods

The fractionation performance of each system was evaluated by calculating the yield and purity. Yield represents the ratio between the mass of a fraction of EPS (polysaccharides or proteins) in one of the phases (bottom, top, or interfacial precipitate) and the initial mass of the respective EPS’s fraction. The yield of EPS’s polysaccharide (*Y*_PS_,_j_ %) was calculated using eq 1, where j represents one of the phases (bottom, top, or interfacial precipitate). Similarly, eq 2 was used to calculate EPS’s protein yield (*Y*_PN_,_j_ %).

The purity was defined as the ratio between the mass of one of the EPS’s fractions (polysaccharides or proteins) and the total mass of the EPS present in the phase. The purity of EPS polysaccharide (Purity_PS,j_ %) was calculated using eq 3, where j represents one of the phases (bottom, top, or interfacial precipitate), while the purity of EPS protein (Purity_PN,j_ %) fraction was calculated based on eq 4.
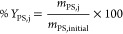
1
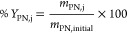
2
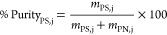
3
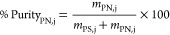
4

## 3 Results and Discussion

### 3.1 Effect of Type of Phase-Forming Compounds

#### 3.1.1 Ionic Liquid-Based TPP

The impact of the nature of the phase-forming compounds was studied to validate which type of TPP systems is suitable for EPS fractionation, that is, alcohol, polymer, or ionic liquid-based. Experiments were carried out with phase-forming compounds at concentrations similar to those used for the model compound studies (as described in Section 2.2). It was confirmed that indeed three phases were also formed with the applied concentrations in the presence of EPS. The detailed data on the yields and purity levels are given in the Supporting Information (Table S1).

For systems composed of IL and phosphate salt, EPS’s polysaccharide (EPS-PS) was preferentially extracted to the IL-rich/top phase (*Y*_PS,top_ = 54–61%). The yield of EPS-PS to the top phase, based on the type of IL, was as follows: [C_4_mim]Cl > [C_4_mim]Br ≈ [C_4_mim][CH_3_CO_2_]. In addition, a substantial amount of EPS-PS was also found as an interfacial precipitate. Still for IL/phosphate-based systems, it was observed that EPS’s protein fraction (EPS-PN) was preferentially precipitated at the interface (*Y*_PN,prec_ = 46–49%) and, to a lesser extent, also migrated to the IL-rich phase. The EPS-PN yields (in bottom, precipitate, and top phases) were not substantially different using imidazolium ILs composed of different anions. It was also observed that none of the EPS’s fractions had preferential affinity for the salt-rich/bottom phase, as low extraction yields (*Y*_bottom_ < 15%) were observed in this phase.

Since EPS-PS and EPS-PN fractions showed preferential affinity for similar phases (that is, the IL-rich phase and precipitate), the IL/K_2_HPO_4_ TPP systems were not selective enough to increase the purity of EPS fractions. As shown in Table S1, the highest purity improvement was obtained for the system composed of [C_4_mim]Br/K_2_HPO_4_ as the purity of EPS-PS and EPS-PN increased by 1.06-fold and 1.32-fold, respectively.

Compared to our previous investigation using the same ILs and phosphate salt for the fractionation of model biopolymers,^41^ a comparable trend was observed based on the type of IL. That is, [C_4_mim]Cl was also the most suitable IL for the fractionation of model biopolymers. In terms of the preferential phase of each type of biopolymer, a divergent trend was observed. For model compounds, it was seen that alginate (polysaccharide) had preferential affinity for the salt-rich phase, while BSA (protein) majorly migrated to the IL-rich phase. Using real EPS samples, both fractions (EPS-PS and EPS-PN) had a preferential affinity for the IL-rich phase and precipitate.

Compared to the literature, both trends were observed, and it is likely due to the structural diversity of the biopolymer investigated. For instance, Tan et al.^66^ reported that the fractionation of the real biopolymer mixture results into the polysaccharide fraction to be mostly in the salt-rich phase, while the majority of proteins partitioned to the IL-rich phase. On the other hand, in the study carried out by Alvarez-Guerra,^53^ accumulation of lactoferrin (protein) at the interface (83–99% yield) was observed in the IL-based system. Similarly, Suarez Ruiz et al.^54^ showed that 94% of proteins from the microalgae mixture were also found as the interfacial precipitate in the PPG400/[Ch][DHP] system.

**Figure 1:**
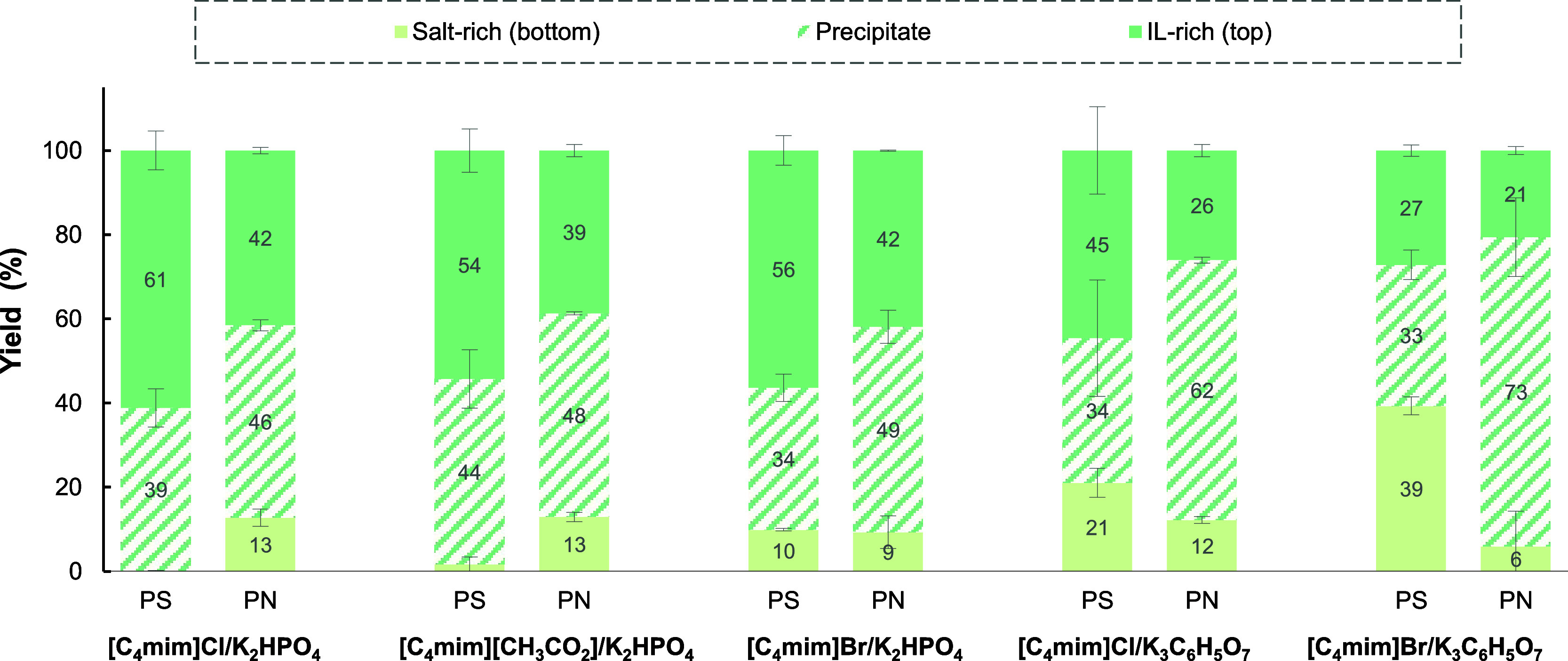
Yields of separation of EPS’s polysaccharide (PS) from EPS’s protein (PN) present in the EPS aqueous extract by [C_4_mim]-based TPP. Systems composed of 15 wt % IL + 25 wt % salt, except citrate-based ones (20 wt % IL + 40 wt % salt).

To improve the selectivity of the system, another salt (citrate) was used as a replacement of phosphate as the phase-forming compound. As shown in Figure 1, for IL/citrate-based TPP systems, the preferential phase for EPS-PS seemed to be dependent on the type of IL. [C_4_mim]Cl showed a similar trend as observed for IL/phosphate-based systems, that is, IL-rich as the preferential phase for EPS-PS (*Y*_PS,top_ = 45%). On the other hand, EPS-PS was mostly found in the salt-rich phase (*Y*_PS,bottom_ 39%) for the [C_4_mim]Br system. Regarding the partitioning behavior of EPS-PN, a higher precipitation of EPS-PN (*Y*_PN,prec_) at the interface was observed for citrate-based systems in relation to phosphate-based ones. It should be noted that due to the highly hydrophilic nature of citrate salt, a higher concentration of salt was required to form the biphasic extraction platform. Hence, the higher precipitation of EPS-PN might be caused by the higher concentration of phase-forming compounds required.

The partitioning behavior of biopolymers in TPP systems is a complex phenomenon and may involve several driving forces, namely, electrostatic interaction, hydrogen bonding interaction, and steric effects.^67^ In this case, EPS-PS preferentially migrated to the IL-rich phase probably due to favorable electrostatic interaction among charged EPS-PS and IL ions. In addition, the preferential precipitation of EPS-PN seems to be related to the reduced solubility of proteins in high ionic strength environments as reported in the literature.^68^

Among the investigated IL-based TPP systems, [C_4_mim]Br/K_3_C_6_H_5_O_7_ had the best performance, as it combined both high yields and purity. This system allowed the recovery of the EPS-PS fraction in the salt-rich phase (*Y*_PS,bottom_ = 39%) with purity of 96%, while the EPS-PN fraction was recovered as the precipitate (*Y*_PN,prec_ = 73%) with purity of 38%. This means that the purity of EPS-PS and EPS-PN fractions improved by 1.06-fold and 1.74-fold, respectively.

#### 3.1.2 Polymer-Based TPP

Polymer-based TPP was also studied for the fractionation of EPS. Systems were composed of polyethylene glycol (PEG) of different molecular weights (400 and 1000 g/mol) combined with different salts (sulfate, phosphate, and citrate). It was also confirmed that the formation of three phases using mixture points was similar to that used in the model compound studies. The detailed data on the yields and purity levels are given in the Supporting Information (Table S2).

**Figure 2:**
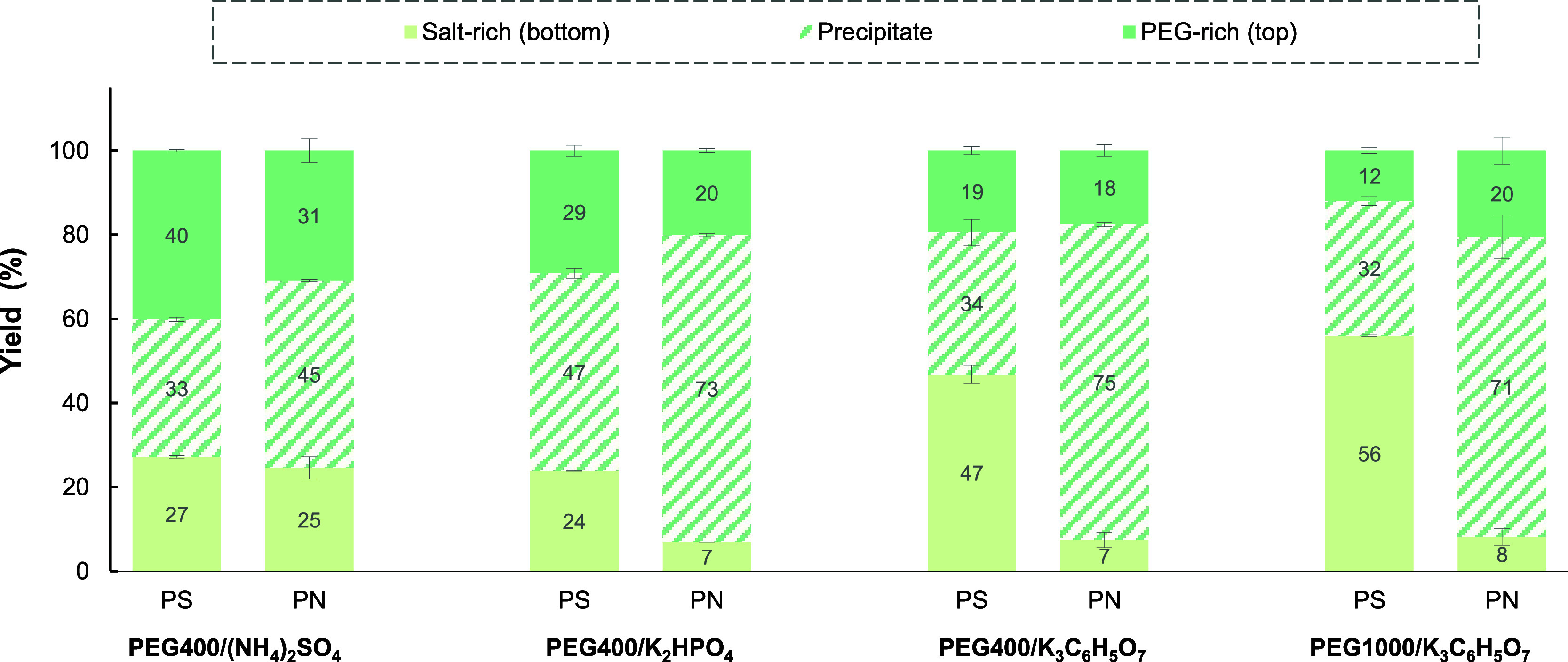
Yields of separation of polysaccharide (PS) from protein (PN) present in EPS by PEG-based TPP. Systems composed of 15 wt % polymer + 25 wt % salt, except citrate-based ones (20 wt % PEG + 30 wt % salt).

For PEG400-based systems, the preferential phase of EPS’s polysaccharide (EPS-PS) depended on the type of salt used (Figure 2). In the PEG 400/(NH_4_)_2_SO_4_ system, EPS-PS mostly partitioned to the PEG-rich/top phase (*Y*_PS,top_ = 40%), while for the PEG400/K_2_HPO_4_ system, EPS-PS was mostly found as the precipitate (*Y*_PS,prec_ = 47%). Lastly, for the PEG400/K_3_C_6_H_5_O_7_ system, EPS-PS mostly partitioned to the salt-rich/bottom phase (*Y*_PS,bottom_ = 47%). The partitioning behavior of EPS’s proteins (PN) was similar for different salts as EPS-PN mostly precipitated at the interface. The precipitation yields of proteins (*Y*_PN,prec_) ranged from 45 to 75% and, based on the type of salt, it was as follows: (NH_4_)_2_SO_4_ < K_2_HPO_4_ ≈ K_3_C_6_H_5_O_7_. It was also observed that the pH of the system depended on the type of salt used. For instance, the use of sulfate salt as the phase-forming compound led to a pH of 6, K_3_C_6_H_5_O_7_ resulted in a pH of 8, and phosphate salt led to a pH of 13. It should be noted that with the current experimental design, it was not possible to draw conclusive insights regarding the effect of pH on EPS partitioning.

Similar to the observation for IL-based TPP systems, using K_3_C_6_H_5_O_7_ as a phase-forming compound also for the PEG-based TPP systems led to the highest yields. Hence, this salt was selected for further investigation, regarding the effect of PEG’s molecular weight. As shown in Figure 2, for the PEG1000/K_3_C_6_H_5_O_7_ system, EPS’s polysaccharide (EPS-PS) also mostly migrated to the salt-rich phase, with a higher yield (*Y*_PS,bottom_ = 56%) in relation to the PEG400-based system (*Y*_PS,bottom_ = 47%). Similar precipitation yield of EPS-PN was observed for systems based on PEG400 (*Y*_PN,prec_ = 75%) and PEG1000 (*Y*_PN,prec_ = 71%).

In terms of selectivity, the purities of EPS-PS and EPS-PN obtained for different phases are shown in Table S2. Based on the type of salt, improvements in the purity of EPS-PS were as follows: K_2_HPO_4_ < (NH_4_)_2_SO_4_ < K_3_C_6_H_5_O_7._ A slightly different trend was observed for EPS-PN, as the (NH_4_)_2_SO_4_-based system was the least selective, and the K_3_C_6_H_5_O_7_-based system remained the most selective. Nevertheless, for both EPS fractions, the highest improvement of purity was obtained for the citrate-based systems, and using either PEG 400 or PEG 1000 did not seem to affect the selectivity of the TPP system.

Compared to our previous investigation using PEG400 and similar salt,^42^ EPS partitioning behavior was considerably distinct from that of model compounds (dextran and BSA). More specifically, it was observed that BSA (model protein) was mostly extracted to the PEG-rich phase (79–98%), while EPS-PN was mostly precipitated at the interface. It is likely that the presence of low molecular weight molecules and humic acids in EPS samples reduced the level of solubilization of EPS-PN into the top phase and induced precipitation. Compared to the literature, using real biopolymer samples has also shown similar partitioning behavior of proteins as observed in this work. In the fractionation of microalgae biomass by PEG400-citrate ATPS/TPP, 77% of the proteins could be recovered as the interfacial precipitate.^54^ Preferential precipitation of IgG at the interface (66%), a biopharmaceutical protein, was also reported for the TPP system composed of PEG1000 and citrate.^69^

Regarding the polysaccharide fraction, EPS-PS showed noticeable yields to PEG-rich phases (*Y*_PS,top_ = 12–40%), while nearly no dextran or alginate (model polysaccharides) was found in the PEG-rich phases. Compared to the literature, similar behavior using real samples was reported by Du et al.^70^ as sulfated polysaccharide, from the EPS crude extract, also preferentially partitioned to the PEG-rich phase. Lastly, it was also observed that the purity of EPS fractions was considerably lower than that of model compound systems,^42^ which was anticipated due to the compositional complexity of EPS samples.

It is known in the literature that polysaccharides usually preferentially migrate to the salt-rich phase in ATPS [24] as this phase is the most hydrated one. In this investigation, by using polymer-based systems, EPS-PS mostly precipitated at the interface and migrated to the top-phase for phosphate- and sulfate-based systems, respectively, and this is likely due to the relatively high salt concentration used to form the fractionation platform (25 wt %), which caused an excessive competition for water molecules in the bottom phase. An exception was observed for citrate-based systems, in which EPS-PS was mostly extracted to the salt-rich phases (*Y*_PS,bottom_ = 47–56%) even though a higher salt concentration was used (30 wt %). The higher partitioning of EPS-PS to the salt-rich phase in citrate-based systems is possibly because of lower water competition as this salt has a lower charge density due to a larger anion and because of its ability to establish hydrogen bonding with hydroxyl groups present in EPS-PS, as opposed to sulfate and phosphate salts. It is also likely that using PEG 1000 improved the extraction of EPS-PS to salt-rich phases due to steric effects. That is, the use of PEG1000 lowered the migration of EPS-PS migrated to the top phase, resulting in higher partition of EPS-PS to the salt-rich phase instead. An increase in hyaluronic acid recovery was reported in the literature as PEG MW increased from 6000 to 8000 g/mol.^71^

Altogether, among the polymer-based TPP systems, PEG1000/K_3_C_6_H_5_O_7_ had the best performance, in terms of fractionation yield and selectivity. For this system, 56% EPS’s polysaccharide migrated to the salt-rich phase (purity = 96%), while 71% EPS’s protein precipitated at the interface (purity = 39%). This means that the purity of EPS-PS and EPS-PN fractions improved by 1.23-fold and 1.76-fold, respectively.

#### 3.1.3 Alcohol-Based TPP

Alcohol-based systems were another class of TPP systems investigated in this work. Sulfate, phosphate, and citrate salts were used as phase-forming compounds, combined with ethanol (EtOH) and 2-propanol (2-PrOH). Similar to the other types of TPP systems, the formation of three phases using mixture points similar to those used in our model compound studies was also confirmed. The detailed data on the yields and purity are given in the Supporting Information (Table S3).

**Figure 3:**
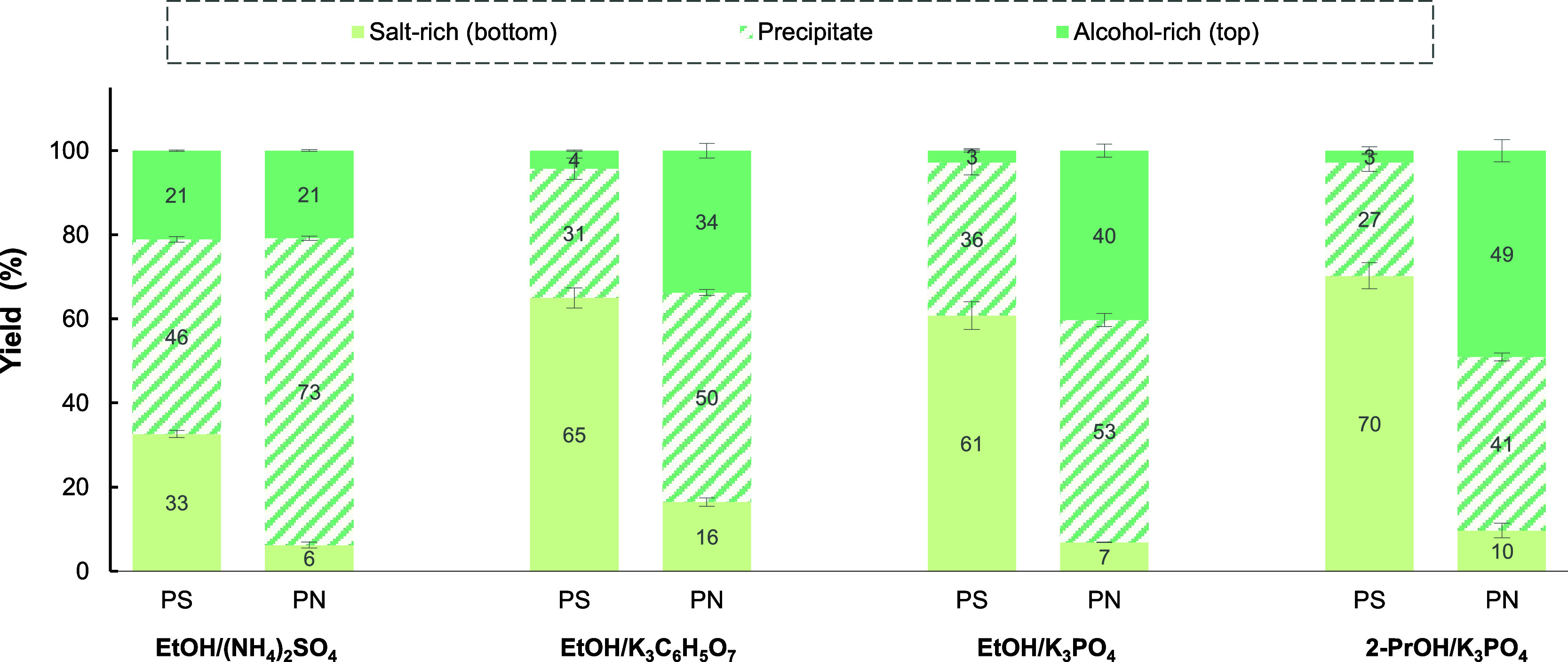
Separation of polysaccharide (PS) from protein (PN) present in EPS by alcohol-based TPP. Systems composed of 30 wt % EtOH + 15 wt % salt, except citrate-based one (34 wt % EtOH + 16 wt % salt).

For systems using ethanol, it was observed that the preferential phase of EPS-PS was the salt-rich phase when using citrate (*Y*_PS,bottom_ = 65%) and phosphate salt (*Y*_PS,bottom_ = 61%), while EPS-PS mostly precipitated when sulfate salt was used (Figure 3). EPS-PN mostly precipitated at the interface for all ethanol-based systems, with yields ranging from 50 to 73%. Regarding the effect of the type of alcohol, using 2-propanol led to a slightly higher EPS-PS yield in the salt-rich phase and lower EPS-PN precipitation yield in relation to ethanol-based systems. It has been reported that the phase separation ability of 2-propanol is higher than ethanol due to stronger intermolecular interactions.^59^ In other words, 2-propanol is more easily excluded from the salt-rich phase to the alcohol-rich phase compared with ethanol, which implies a higher solubility of EPS-PS into the salt-rich phase for this type of TPP system. Similar to what was observed for polymer-based TPP, the pH of the system depended on the type of salt used. For instance, the use of sulfate salt as the phase-forming compound led to a pH of 6, citrate resulted in a pH of 8, and phosphate salt led to a pH of 13. For this system, the current experimental design also limited the understanding about the effect of pH on EPS partitioning.

Regarding the selectivity of alcohol-based TPP systems, the purities of EPS-PS and EPS-PN obtained for different phases are shown in Table S3. High purity EPS-PS was obtained in the bottom phases (salt-rich), ranging from 93 to 97%, which represented an improvement of 1.20 to 1.24-fold in relation to the initial feed concentration of EPS-PS. Regarding the purity of the EPS-PN fraction, it was observed that for citrate- and phosphate-based systems, even though high purity was obtained in the top phase (69–83%), lower yields were observed for this phase, which favors the recovery of EPS-PN from precipitate. It should be noted that in some cases, high yields in the precipitate are advantageous because it implies a simpler regeneration process after fractionation. Precipitation can also be considered disadvantageous as it was in our previous investigation,^41^ where interfacial precipitation was also observed when using extractive systems based on phosphonium ionic liquids, and this precipitate was not soluble in a variety of solvents, making the biopolymer recovery not feasible. In this case, the precipitate could be readily solubilized in aqueous media, making biopolymer recovery possible. Hence, based on the recovery of EPS-PN from the precipitate phase, the highest improvement of purity (for both EPS fractions) was obtained for the systems composed of EtOH/K_3_C_6_H_5_O_7_ as the purity of EPS-PS and EPS-PN increased approximately around by 1.20-fold and 1.43-fold as compared to the initial feed concentration of EPS-PS and EPS-PN, respectively.

Compared to our previous investigation using model compounds,^42^ it was observed that the anionic model polysaccharides (alginate and gum arabic) were mostly extracted to the salt-rich phases. The same trend was observed for EPS-PS for phosphate and citrate salts and already expected since Ajao et al.^17^ reported the presence of carboxyl group in the EPS backbone. The presence of charge enhances polysaccharide’s affinity for water,^72^ which leads to their preferential partition toward the most hydrated phase (that is, salt-rich phase). Regarding the partitioning behavior of the protein, it was observed that different model proteins had different preferential phases. BSA was mostly extracted to the alcohol-rich phase, while lysozyme mostly precipitated in ethanol-based TPP systems. Compared to the literature, the commonly reported TPP system (composed of butanol and sulfate salt) also shows precipitation of proteins at the interface.^73^ In addition, Jiang et al.^48^ also reported that the polysaccharide fraction of the real EPS mixture obtained from lactic acid bacteria mostly partitioned to the salt-rich phase (yield at 75%) in an ethanol-based TPP system. It should be noted that, compared to commonly reported TPP systems (such as the ones based on ammonium sulfate and butanol),^73^ using ethanol as the phase-forming compound is more beneficial as it is recognized as a less toxic solvent^74^ and because of its lower boiling point, which reduces the energy demand in potential recycling processes involving evaporation. In addition, the replacement of ammonium sulfate for a biodegradable salt (potassium citrate) is also advantageous from an environmental point of view.

Concluding, the results showed that EPS-PN mostly precipitated at the interface for EtOH systems, and it might be attributed to protein solubility being affected by ions. At low ion concentrations (<0.5 M), protein solubility increases along with ionic strength. This effect is known as “salting-in”, and it occurs because ions in the solution shield protein molecules from the charge of other protein molecules, hence preventing aggregation. At a very high ionic strength, the surface of the protein will become so charged that once again protein solubility decreases as ionic strength increases, known as the “salting-out” effect.^68^ In addition, polysaccharides preferentially migrate to the salt-rich phase in ATPS composed of polymer/IL/alcohol and (in)organic salt^24^ as this phase is the most hydrated phase in the system, enabling the solubilization of such highly hydrophilic macromolecules. The obtained results followed this trend, except for the sulfate-based system. This probably occurred because ethanol is less excluded from the salt-rich phase toward the ethanol-rich phase since (NH_4_)_2_SO_4_ is a weaker salting-out agent than phosphate and citrate salts. As a result, a higher concentration of ethanol in the bottom phase seemed to occur in the sulfate-based system, reducing the solubility of EPS-PS.

Altogether, among alcohol-based TPP systems, EtOH/K_3_C_6_H_5_O_7_ had the best performance in terms of yield and selectivity for both EPS fractions. 65% EPS-PS partitioned to the salt-rich/bottom phase (purity = 93%), and 50% EPS-PN was enriched at the interface (purity = 31%).

### 3.2 Effect of Concentration of Phase-Forming Compounds

As shown in Section 3.1, TPP systems composed of alcohol or polymer in combination with citrate salt demonstrated to be potential separation platforms for the fractionation of EPS. Such systems were further investigated, regarding the effect of the concentration of phase-forming compounds on their performance so that reduction of the mass of phase-forming compounds required can be achieved without loss of yield and selectivity.

**Figure 4:**
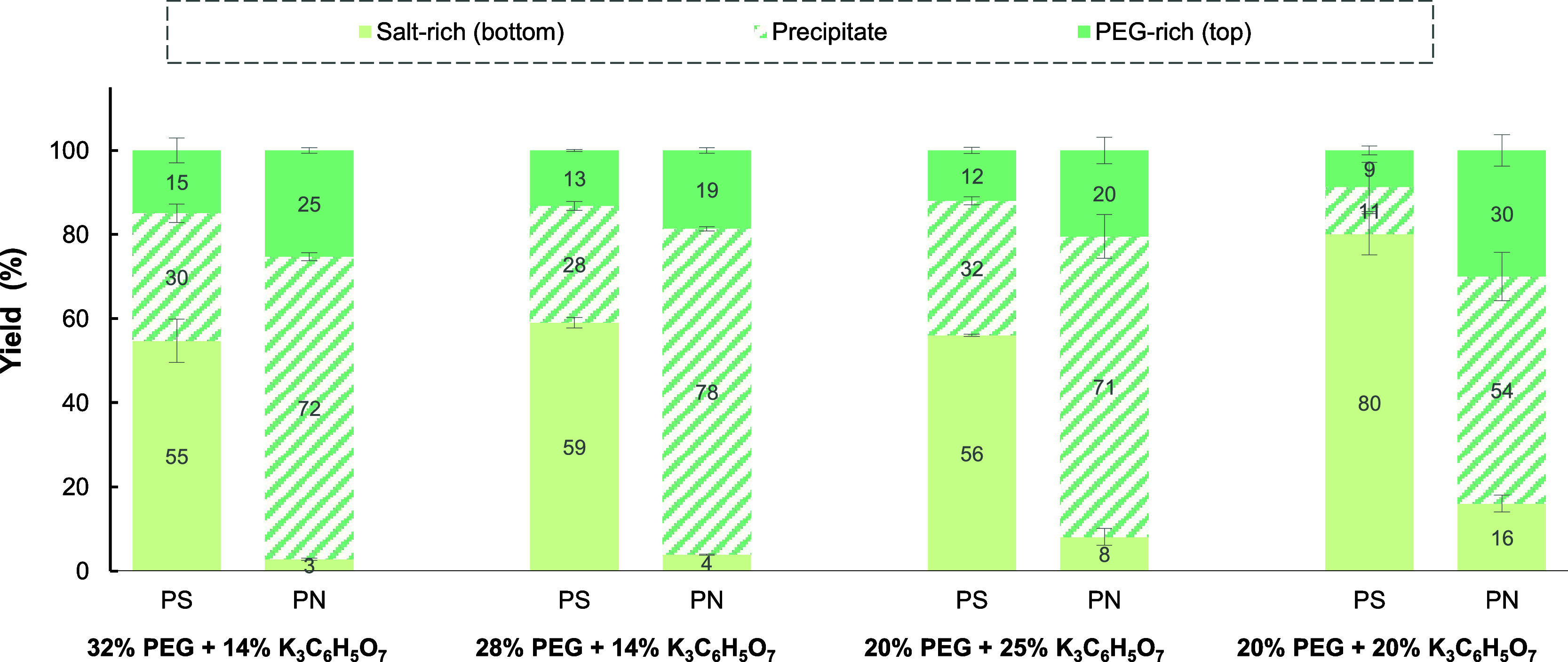
Effect of the concentration of PEG1000 and K_3_C_6_H_5_O_7_ (expressed in w/w %) on the separation of polysaccharide and protein present in the EPS mixture by PEG/citrate TPP.

For polymer-based TPP systems, different concentrations of PEG1000 (20, 28, and 32 wt %) and K_3_C_6_H_5_O_7_ (14, 20, and 25 wt %) were evaluated. The detailed data on the yields and purity levels are given in the Supporting Information (Table S4). As shown in Figure 4, for all mixture points, fractionation was achieved as EPS-PS mostly partitioned to the salt-rich/bottom phase, while EPS-PN was obtained as the interfacial precipitate. For the different mixture points_,_ yields of EPS-PS (*Y*_PS,bottom_) and EPS-PN (*Y*_PN,precipitate_) ranged from 55 to 80 and 54–78%, respectively. It was also observed that reducing the concentration of PEG1000 from 32 to 20 wt % did not considerably change the yield of EPS-PS and EPS-PN. On the other hand, the concentration of K_3_C_6_H_5_O_7_ seemed to play a more relevant role in TPP’s performance. Reducing the concentration of salt from 25 to 20 wt % increased EPS-PS yield (*Y*_PS,bottom_) from 56 to 80%, respectively. This represents an increase of 1.43-fold in the yield of polysaccharides to the salt-rich phase (*Y*_PS,bottom_). It should be noted that reducing citrate concentration also increased migration of EPS-PN to the salt-rich phase, causing an unfavorable reduction of the protein retrieved as the precipitate.

Regarding the effect of phase-forming compounds on selectivity (Table S4), it was observed that using different mixture points did not have a relevant impact for the EPS-PS fraction as the purity of this fraction ranged from 96 to 98%. For EPS-PN, it was observed that changes in the concentration of citrate salt affected its purity as decreasing salt concentration from 25 wt % to 20 wt % increased the purity of EPS-PN in the precipitate from 39 to 54%. Similar to what was observed for yield, the selectivity of TPP systems seemed to be more affected by changes in citrate concentration.

Changes in the concentration of the phase-forming compounds imply changes in critical factors associated with the solubilization of biopolymers, namely, the water content of the phases and the competition between phase-forming compounds and biopolymers for water molecules. The best performance in terms of yield and purity was obtained using a relatively higher concentration of PEG, in relation to citrate salt, because it ensured limited partitioning of EPS-PN to the polymer-rich phase (due to steric effects) while maintaining proper solubilization EPS-PS into the salt-rich phase due to less competition of salt ions for water molecules. Compared to our previous investigation, the observed trends are in agreement as the highest partitioning of BSA (model protein) to the polymer-rich phase occurred at the lowest concentration of PEG.^42^ Compared to the literature, Jiang et al.^75^ also reported that excessive salt competition for water molecules in the bottom phase led to reduced solubility of polysaccharides in the bottom phase in the PEG600/Na_2_HPO_4_ system. It should be noted that, compared to commonly reported TPP based on (NH_4_)_2_SO_4_/butanol,^76^ polymer-based TPP systems present comparable performance and represent a potential alternative to the fractionation of biomolecules which are incompatible with alcohol-based systems.

Overall, the best performance was achieved for the mixture point with 28 wt % PEG1000 + 14 wt % K_3_C_6_H_5_O_7,_ as 59% EPS-PS partitioned to the salt-rich phase (purity = 98%), while 78% of EPS-PN could be retrieved in a concentrated form as precipitate (purity = 44%). These results led to increases of 1.26 and 2.00-fold in the purity of EPS-PS and EPS-PN, respectively.

**Figure 5:**
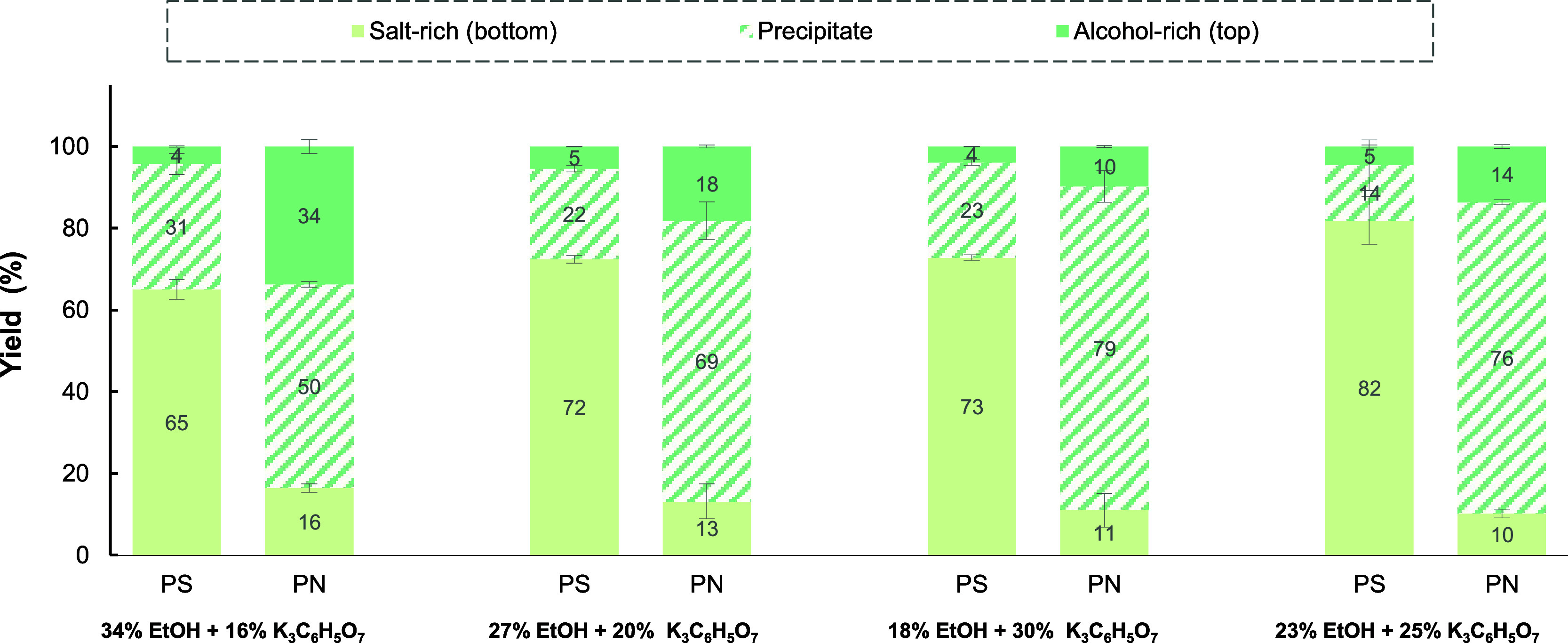
Effect of the concentration of EtOH and K_3_C_6_H_5_O_7_ (expressed in w/w %) on the separation of polysaccharide and protein present in EPS mixture by EtOH/K_3_C_6_H_5_O_7_ TPP.

Similarly, the performance of alcohol-based TPP systems was evaluated using different concentrations of ethanol and K_3_C_6_H_5_O_7,_ as shown in Figure 5 and in detail in the Supporting Information (Table S5). The yield of EPS-PS to the salt-rich phase (*Y*_PS,bottom_) increased from 73 to 82% as citrate concentration decreased from 30 to 25 wt %. Further decrease in salt concentration up to 16 wt % decreased the yield (*Y*_PS,bottom_ = 65%) as a result of more EPS-PS precipitation at the interface. The highest yield was obtained for the system composed of 23 wt % EtOH + 25 wt % K_3_C_6_H_5_O_7_. EPS-PN was mostly obtained as a precipitate, with yield ranging from 50 to 79%. It was observed that increasing the concentration of citrate, while simultaneously reducing the concentration of ethanol, improved the precipitation of EPS-PS. In other words, the highest precipitation yield (*Y*_PN,prec_ = 79%) was found for a mixture point with 18 wt % EtOH + 30 wt % K_3_C_6_H_5_O_7_, while the lowest precipitation yields of EPS-PN (*Y*_PN,prec_ = 50%) occurred for a mixture point with 34 wt % EtOH + 16 wt % K_3_C_6_H_5_O_7._

Regarding selectivity (Table S5), it was observed that the highest purity improvement, for both fractions, was obtained for the mixture point with 23 wt % EtOH + 25 wt % K_3_C_6_H_5_O_7_. Other systems had a lower improvement in purity due to undesirable precipitation of EPS-PS or solubilization of EPS-PN in the PEG-rich/top phase.

The observed trend for partitioning of EPS-PS can be understood considering that as salt concentration decreased, there was less competition between salt and polysaccharides for water molecules, which improved the partitioning of EPS-PS to the salt-rich phase. It is also worth noting that decreasing salt concentration is also implied in using a higher concentration of alcohol as the phase-forming compound. Consequently, as salt concentration decreased, while increasing the alcohol concentration, a preferential migration of water molecules occurred toward the alcohol-rich phase, which resulted in less polysaccharides extracted to the salt-rich phase. Altogether, optimal EPS-PS yields can be obtained keeping the salt concentration low enough to allow sufficient solubilization of polysaccharides in the salt-rich phase and high enough to maintain appropriate water content in the bottom phase due to competition with alcohol molecules.

Regarding the partitioning of EPS-PN, it was observed that using a higher concentration of ethanol in relation to citrate salt increased the solubility of EPS-PN into the alcohol-rich phase because that promotes the preferential migration of water molecules to the alcohol-rich phase.^77^ As more salt was added to the system, the water content in the alcohol phase decreased, which promoted the precipitation of EPS-PN at the interface. Hence, EPS-PN yields benefit from salt concentrations high enough to maintain a relatively low water content in the ethanol-rich/top phase. Compared to the literature, a similar observation, regarding water content in the phase and the relative concentration of phase-forming compounds, has been reported by Jiang et al.^48^ for the fractionation of EPS obtained by single microbial culture. The authors found that as (NH_4_)_2_SO_4_ concentration exceeded 18 wt %, the ability of the salt to capture water molecules was stronger than that of ethanol, resulting in a higher partition of EPS to the salt-rich phase.

Altogether, the best fractionation performance was obtained for the system composed of 23 wt % EtOH + 25 wt % K_3_C_6_H_6_O_7_, as it was possible to obtain 82% EPS-PS in the salt-rich phase (purity = 97%), while 76% EPS-PN was retrieved as precipitate (purity = 62%). This represents an increase of 1.24- and 2.83-fold in the purity of EPS-PS and EPS-PN, respectively, as compared to the initial feed concentration. Compared to TPP systems generally reported in the literature, the ethanol/citrate systems investigated in this study showed comparable performance for simultaneous separation of proteins and polysaccharides from the real biopolymer mixture while requiring a lower amount of phase-forming compounds and relies on a biodegradable salt.^76,78^

### 3.3 Isolation of Fractionated EPS and Regeneration of Phase-Forming Compounds

**Figure 6:**
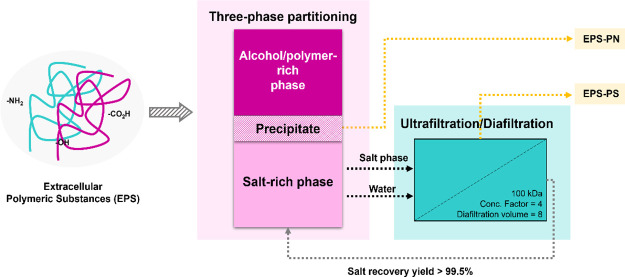
Scheme of the fractionation process of EPS by a three-phase partitioning system combined with ultrafiltration/diafiltration.

Figure 6 shows an overview of the process of the fractionation of EPS. After the fractionation step, the following step is the separation of the targeted biopolymer (EPS-PS and EPS-PN) from phase-forming compounds used in TPP systems. The approach to achieve such separation depends on the phase to be considered. For instance, EPS-PN was recovered as the interfacial precipitate. EPS-PN contents in the precipitate were 63 and 48 w/w% for the best performing PEG- and EtOH-based systems, respectively. This represents an increase of 2.86- and 2.18-fold on EPS-PN content. The bottom phase (salt-rich) is the preferential phase for the EPS’s polysaccharide fraction. To separate polysaccharides from salt, both ultrafiltration and precipitation techniques were investigated. Preliminary evaluation of precipitation as separation technique showed that by using methanol as the antisolvent (*S*/*F* ratio 2:1, *T* = 21 °C ± 1), 86% (±4.03) EPS-PS could be retrieved from the salt-rich phase. Removal of methanol, by means of evaporation, is still required to enable the recycling of salt stream to form a new extractive platform, which increases the complexity of the process.

Alternatively, ultrafiltration operated in the diafiltration mode (UF/DF) has already been confirmed by previous investigation to be able to efficiently separate model biopolymers from the compounds used in TPP systems.^41,42^ As shown in Table 2, UF/DF allowed isolation and concentration of EPS-PS (retentate stream) as well as regeneration of phase-forming compounds (permeate stream) in a single stage. Citrate recovery (>99%) was similar for both bottom phases used in the filtration assays (PEG-based and EtOH-based systems).

**Table 2:** Recovery Yields of EPS-PS and K_3_C_6_H_5_O_7_ after Ultrafiltration/Diafiltration^a^

three-partitioning system	EPS-PS initial (mg/mL)	EPS-PS final (mg/mL)	K_3_C_6_H_5_O_7_ recovery (%)	EPS-PS recovery (%)	EPS-PS content (w/w %)	EPS-PN content (w/w%)
28 wt % PEG1000/14 wt % K_3_C_6_H_5_O_7_	2.62	8.48	99.50	101.97	87.09	0.65
23 wt % EtOH/25 wt % K_3_C_6_H_5_O_7_	1.09	3.06	99.76	85.05	72.88	0.63

a Feed was the bottom phase obtained from PEG-based and EtOH-based three-phase partitioning systems. Operational conditions: T = 20 °C, 3 bar, diafiltration volume = 8.

A comparable salt recovery yield was observed in previous work on the separation of phase-forming compounds from BSA (model protein) by ultrafiltration.^42^ For the recovery of EPS-PS, it was observed that about 15% of EPS-PS was lost during filtration of the bottom phase from the EtOH/K_3_C_6_H_5_O_7_ system. When compared to model systems, the separation of the EPS fraction from phase-forming compounds led to higher loss of biopolymer.^42^ This finding is likely because filtration promoted not only the increase in concentration of EPS-PS but also the phase-forming compounds present in the feed. Hence, the increased concentration of salt and alcohol seemed to decrease EPS-PS solubility in the aqueous feed and, consequently, precipitation onto the membrane surface. Compared to the model biopolymer, EPS-PS seemed more prone to precipitation likely due to the higher molecular weight (MW). Ajao et al.^17^ reported EPS’s average molecular weight as 1000 kDa, which is about 2–3 times larger than the model biopolymer’s MW.^42^ Nevertheless, for both systems, it was possible to obtain EPS-PS with low protein content (<1 w/w %), which supports the potential of TPP systems in combination with UF/DF for fractionation of EPS.

## 4 Conclusions

The findings indicated that TPP systems composed of ethanol or PEG, in combination with salts, showed promising results for the fractionation of real EPS. For the alcohol-based TPP systems, the best performance was achieved for the system composed of 23 wt % EtOH and 25 wt % K_3_C_6_H_5_O_7_ as it was possible to obtain 82% EPS-PS in the salt-rich phase, while 76% EPS-PN was retrieved as the precipitate. This represented an increase of 1.24- and 2.83-fold in the purity of EPS-PS and EPS-PN, respectively. For the polymer-based TPP, the system composed of 28 wt % PEG1000 and 14 wt % K_3_C_6_H_5_O_7_ displayed the best yields and selectivity. In this case, 59% EPS-PS partitioned to the salt-rich phase, and 78% EPS-PN could be retrieved in a concentrated form as the precipitate. These results led to an increase of 1.26 and 2.00-fold in the purity of EPS-PS and EPS-PN, respectively. Overall, the ethanol-citrate TPP system combined both high yield and selectivity, being the most promising platform for EPS fractionation. These results confirm that alternative phase-forming compounds can be used other than ammonium sulfate and t-butanol as traditionally proposed in the literature. In addition, PEG-citrate TPP also showed to be a potential alternative to fractionation biopolymer mixtures incompatible with alcohol.

Regarding the recyclability of phase-forming compounds, the ultrafiltration/diafiltration mode was found to be a feasible technique to recover the targeted EPS fraction from phase-forming compounds. Using diafiltration volume (DV) at 8, > 99% of salt present in the feed could be recovered in the permeate stream. EPS-PS fraction was recovered in the retentate phase in a concentrated form with low protein content (<1 w/w%). It should be noted that the salt content in the EPS-PS fraction still remained substantial (12–27 w/w %), so additional filtration steps are still required to improve the overall purity of EPS-PS. On the other hand, EPS-PN was recovered directly from the interface as a precipitate, with protein content ranging from 48 to 63 w/w% (based on the total mass of biopolymer). While this represents an improvement of 2.18- to 2.86-fold in EPS-PN purity in relation to its initial feed concentration, additional fractionations steps are likely to be needed to explore more value-added applications for protein fraction from EPS matrix.

While this study has made significant contributions by exploring a cost-effective, scalable approach for EPS fractionation, some knowledge gaps remain to be addressed. While only the effect of concentration and type of phase-forming compounds were considered in this investigation, pH is also another relevant variable when it comes to biopolymer separation, Hence, gaining understanding on how different pH environments impact EPS fractionation by TPP systems as well as EPS’s protein stability is still necessary. One of the gaps is that this investigation was carried out on a lab scale as it primarily served as a proof-of-concept for a high-throughput fractionation technique for EPS. To fully realize the potential of this approach, further research is necessary to bridge the gap between small-scale operations and eventual pilot- and commercial-scale applications. Moreover, additional optimization studies are essential to lower even further the amount of phase-forming compounds required, which is beneficial from an economic and environmental point of view.

The recyclability of the chemicals used is also an aspect that requires attention. In this study, ultrafiltration allowed high recovery of the phase-forming compound as a diluted stream. Hence, additional concentration steps, using techniques such as membrane processes or distillation, should be assessed to evaluate their performance, particularly in terms of energy demand and ease of water reuse. Exploring strategies to mitigate EPS loss during filtration is also needed. Such approaches can be using different operational conditions and varying membrane properties, namely, molecular weight cutoff (MWCO) and material. Furthermore, the performance of TPP using recycled phase-forming compounds still needs to be thoroughly evaluated to ensure no loss of yield and selectivity. Lastly, characterizing the fractionated EPS (in terms of molecular weight, structure, and rheological properties) is still needed. While TPP systems has been demonstrated to be effective in separating polysaccharides from proteins in the EPS broth, its potential on fractionating EPS based on molecular weight or functional groups (e.g., separating neutral polysaccharides from anionic ones) remains less understood. Hence, the characterization of the fractions can contribute to extend the understanding about the range of applications of TPP systems. Such a study is also crucial to unlock EPS’s potential applications. Some potential added-value applications, as already notably proposed in the literature, is related to the fabrication of biopolymer-based composite materials.^79^

Despite its limitations, this study offers valuable insights into the downstream processing of real biopolymer matrices. Such findings can significantly contribute to the development of cost-effective methods for downstream processing of microbial natural polymers and, consequently, promote the utilization of sustainable chemicals in various applications.

## Supporting Information Available

Tables S1–S3: Yields and purity of EPS-PS and EPS-PN fractionated by ionic liquid-, polyethylene glycol- and alcohol-based by three-phase partitioning systems using different types of salt; Tables S4–S5: Yields and purity of EPS-PS and EPS-PN fractionated by polyethylene glycol- and alcohol-based three-phase partitioning systems at different concentrations (PDF)
