# Supplementary material for: Fractionation of Extracellular Polymeric Substances by Aqueous Three-Phase Partitioning Systems
Source: Ind Eng Chem Res. 2024 Jun 6;63(24):10748–60. doi: 10.1021/acs.iecr.4c00840 (PMC11191973; doi:10.1021/acs.iecr.4c00840)
Supplement: Supplementary file 1 — ie4c00840_si_001.pdf [file ie4c00840_si_001.pdf]

Supporting Information

Fractionation of extracellular polymeric substances by aqueous three-phase partitioning systems

Evelyn C. Antunes [a, c], *Bruna Cintra* [a, ‡], *Matthieu Bredel* [a, ‡], *Hardy Temmink* [a, b, †], *Boelo Schuur* [c, *]

(a) Wetsus – European Centre of Excellence for Sustainable Water Technology, Oostergoweg 9, 8911MA Leeuwarden, the Netherlands

(b) Wageningen University and Research, Department of Environmental Technology, Bornse Weilanden 9, 6708 WG, Wageningen, the Netherlands

(c) University of Twente, Sustainable Process Technology Group, Department of Chemical Engineering, Faculty of Science and Technology, Drienerlolaan 5, 7522 NB Enschede, the Netherlands

‡These authors contributed equally

†Hardy Temmink sadly passed away on 8 April 2022

* Corresponding author: b.schuur@utwente.nl

This document contains five tables S1 – S5

Table of Contents

1. Effect of the type of three-phase partitioning systems for EPS fractionation 3
1.1. Ionic liquid Three Phase Partitioning 3
1.2. Polymer-based Three Phase Partitioning 4
1.3. Alcohol-based Three Phase Partitioning 5
2. Effect of concentration of phase-forming compounds on TPP performance 6
2.1. Polymer-based Three Phase Partitioning 6
2.2. Alcohol-based Three Phase Partitioning 7

1. Effect of the type of three-phase partitioning systems for EPS fractionation

1.1. Ionic liquid Three Phase Partitioning

Table S1: Yields and purity of EPS-PS and EPS-PN fractionated by $[C_4mim]$-based TPP. Results reported as the average of two independent assays with their respective standard deviation. n/a = not applicable, * = not possible to accurately measure as observed volume of precipitate was most of the cases < 0.125 mL

System	Phase	Volume (mL)	EPS_{PS} (mg)	EPS_{PN} (mg)	Yield EPS_{PS} (%)	Yield EPS_{PN} (%)	Purity EPS_{PS} (%)	Purity EPS_{PN} (%)
15 wt% $[C_4mim]Cl$	Initial	n/a	1.87 (±0.00)	0.53(±0.00)	n/a	n/a	78.00(±0.00)	22.00(±0.00)
25 wt% K_2HPO_4	Bottom	1.75	0.00 (±0.00)	0.07(±0.01)	0.14(±0.08)	12.73(±2.08)	3.89(±2.72)	96.11(±2.72)
	Top	1.25	1.15 (±0.09)	0.22(±0.00)	61.14(±4.61)	41.52(±0.75)	83.89(±1.26)	16.11(±1.26)
	Precipitate*	---	0.73 (±0.09)	0.24(±0.01)	38.72(±4.53)	45.74(±1.33)	74.93(±1.66)	25.07(±1.66)
15 wt% $[C_4mim][CH_3CO_2]$	Initial	n/a	1.87(±0.00)	0.53(±0.00)	n/a	n/a	78.00(±0.00)	22.00(±0.00)
25 wt% K_2HPO_4	Bottom	1.75	0.03(±0.03)	0.07(±0.01)	1.61(±1.80)	12.93(±1.08)	25.88(±23.82)	74.12(±23.82)
	Top	1.25	1.02(±0.10)	0.20(±0.01)	54.29(±5.14)	38.70(±1.43)	83.19(±1.84)	16.81(±1.84)
	Precipitate*	---	0.83(±0.13)	0.26(±0.00)	44.10(±6.94)	48.37(±0.34)	76.20(±2.99)	23.80(±2.99)
15 wt% $[C_4mim][Br]$	Initial	n/a	1.87(±0.00)	0.53(±0.00)	n/a	n/a	78.00(±0.00)	22.00(±0.00)
25 wt% K_2HPO_4	Bottom	2.40	0.18(±0.00)	0.05(±0.02)	9.89(±0.27)	9.29(±3.87)	79.39(±6.48)	20.61(±6.48)
	Top	1.10	1.05(±0.07)	0.22(±0.00)	56.36(±3.52)	41.89(±0.13)	82.65(±0.94)	17.35(±0.94)
	Precipitate*	---	0.63(±0.06)	0.26(±0.02)	33.76(±3.25)	48.82(±3.99)	70.95(±3.67)	29.05(±3.67)
20 wt% $[C_4mim][Cl]$	Initial	n/a	1.25(±0.00)	0.35(±0.00)	n/a	n/a	78.00(±0.00)	22.00(±0.00)
40 wt% $K_3C_5H_6O_7$	Bottom	2.00	0.26(±0.04)	0.04(±0.00)	21.02(±3.48)	12.19(±0.77)	85.86(±1.25)	14.14(±1.25)
	Top	1.00	0.56(±0.13)	0.09(±0.00)	44.56(±10.37)	26.05(±1.43)	85.50(±3.56)	14.50(±3.56)
	Precipitate*	---	0.43(±0.17)	0.22(±0.00)	34.41(±13.85)	61.76(±0.66)	65.13(±9.55)	34.87(±9.55)
20 wt% $[C_4mim][Br]$	Initial	n/a	1.25(±0.00)	0.35(±0.00)	n/a	n/a	78.00(±0.00)	22.00(±0.00)
40 wt% $K_3C_5H_6O_7$	Bottom	2.40	0.49(±0.03)	0.02(±0.03)	39.38(±2.15)	5.94(±8.40)	96.21(±5.35)	3.79(±5.34)
	Top	1.00	0.34(±0.02)	0.07(±0.00)	27.17(±1.33)	20.58(±0.95)	82.40(±0.04)	17.60(±0.04)
	Precipitate*	---	0.42(±0.04)	0.26(±0.03)	33.45(±3.48)	73.49(±9.34)	61.77(±0.55)	38.23(±0.55)

1.2.Polymer-based Three Phase Partitioning

Table S2: Yields and purity of EPS-PS and EPS-PN fractionated by PEG-based TPP. Results reported as the average of two independent assays with their respective standard deviation. n/a = not applicable, * = not possible to accurately measure as observed volume of precipitate was most of the cases < 0.125 mL

System	Phase	Volume (mL)	EPS_{PS} (mg)	EPS_{PN} (mg)	Yield EPS_{PS} (%)	Yield EPS_{PN} (%)	Purity EPS_{PS} (%)	Purity EPS_{PN} (%)
15 wt% PEG400	Initial	n/a	1.87(±0.00)	0.53(±0.00)	n/a	n/a	78.00(±0.00)	22.00(±0.00)
25 wt% $(NH_4)_2SO_4$	Bottom	3.00	0.51(±0.00)	0.13(±0.01)	27.14(±0.27)	24.51(±2.60)	79.71(±1.88)	20.29(±1.88)
	Top	1.25	0.75(±0.00)	0.16(±0.01)	40.15(±0.28)	30.93(±2.83)	82.16(±1.24)	17.84(±1.24)
	Precipitate*	---	0.61(±0.01)	0.24(±0.00)	32.71(±0.56)	44.56(±0.24)	72.24(±0.24)	27.76(±0.24)
15 wt% PEG400	Initial	n/a	1.87(±0.00)	0.53(±0.00)	n/a	n/a	78.00(±0.00)	22.00(±0.00)
25 wt% K_2HPO_4	Bottom	2.50	0.45(±0.00)	0.04(±0.00)	23.85(±0.13)	6.93(±0.05)	92.43(±0.01)	7.57(±0.01)
	Top	1.50	0.55(±0.02)	0.11(±0.00)	29.16(±1.28)	20.09(±0.48)	83.72(±0.27)	16.28(±0.27)
	Precipitate*	---	0.88(±0.02)	0.39(±0.00)	46.99(±1.15)	72.98(±0.43)	69.54(±0.39)	30.46(±0.39)
20 wt% PEG400	Initial	n/a	1.56(±0.00)	0.44(±0.00)	n/a	n/a	78.00(±0.00)	22.00(±0.00)
30 wt% $K_3C_6H_5O_7$	Bottom	2.00	0.73(±0.03)	0.03(±0.01)	46.83(±2.17)	7.38(±1.85)	95.76(±0.84)	4.24(±0.84)
	Top	1.25	0.30(±0.02)	0.08(±0.01)	19.45(±0.98)	17.56(±1.36)	79.68(±2.07)	20.32(±2.07)
	Precipitate*	---	0.53(±0.05)	0.33(±0.00)	33.72(±3.15)	75.06(±0.50)	61.37(±2.06)	38.63(±2.06)
20 wt% PEG1000	Initial	n/a	1.56(±0.00)	0.44(±0.00)	n/a	n/a	78.00(±0.00)	22.00(±0.00)
30 wt% $K_3C_6H_5O_7$	Bottom	2.50	0.88(±0.00)	0.04(±0.01)	56.02(±0.25)	8.12(±2.00)	96.08(±0.95)	3.92(±0.95)
	Top	2.00	0.19(±0.01)	0.09(±0.01)	11.99(±0.74)	20.44(±3.17)	67.56(±4.75)	32.44(±4.75)
	Precipitate*	---	0.50(±0.01)	0.31(±0.02)	31.99(±0.98)	71.45(±5.18)	61.36(±2.44)	38.64(±2.44)

1.3. Alcohol-based Three Phase Partitioning

Table S3: Yields and purity of EPS-PS and EPS-PN fractionated by alcohol-based TPP. Results reported as the average of two independent assays with their respective standard deviation. n/a = not applicable, * = not possible to accurately measure as observed volume of precipitate was most of the cases < 0.125 mL

System	Phase	Volume (mL)	EPS_{PS} (mg)	EPS_{PN} (mg)	Yield EPS_{PS} (%)	Yield EPS_{PN} (%)	Purity EPS_{PS} (%)	Purity EPS_{PN} (%)
34 wt% EtOH	Initial	n/a	1.95(±0.00)	0.55(±0.00)	n/a	n/a	78.00(±0.00)	22.00(±0.00)
16 wt% $K_3C_6H_5O_7$	Bottom	1.50	1.27(±0.05)	0.09(±0.01)	65.00(±2.40)	16.44(±1.01)	93.34(±0.15)	6.66(±0.15)
	Top	3.30	0.08(±0.00)	0.19(±0.01)	4.27(±0.15)	33.76(±1.70)	30.99(±1.82)	69.01(±1.82)
	Precipitate*	---	0.60(±0.05)	0.27(±0.00)	30.73(±2.55)	49.80(±0.70)	68.58(±2.09)	31.42(±2.09)
30 wt% EtOH	Initial	n/a	2.15(±0.00)	0.61(±0.00)	n/a	n/a	78.00(±0.00)	22.00(±0.00)
15 wt% $(NH_4)_2SO_4$	Bottom	1.75	0.70(±0.02)	0.04(±0.00)	32.66(±0.85)	6.21(±0.71)	94.90(±0.68)	5.10(±0.68)
	Top	3.15	0.45(±0.00)	0.13(±0.00)	21.10(±0.20)	20.82(±0.20)	78.22(±0.32)	21.78(±0.32)
	Precipitate*	---	0.99(±0.01)	0.44(±0.00)	46.24(±0.66)	72.96(±0.51)	69.20(±0.45)	30.80(±0.45)
30 wt% EtOH	Initial	n/a	1.72(±0.00)	0.49(±0.00)	n/a	n/a	78.00(±0.00)	22.00(±0.00)
15 wt% K_3PO_4	Bottom	1.80	1.05(±0.06)	0.03(±0.00)	60.78(±3.33)	6.92(±0.05)	96.89(±0.19)	3.11(±0.19)
	Top	3.15	0.05(±0.01)	0.20(±0.01)	2.82(±0.40)	40.29(±1.58)	19.88(±2.87)	80.12(±2.87)
	Precipitate*	---	0.63(±0.05)	0.26(±0.01)	36.40(±2.94)	52.80(±1.53)	70.93(±1.07)	29.07(±1.07)
30 wt% 2-PrOH	Initial	n/a	1.84(±0.00)	0.52(±0.00)	n/a	n/a	78.00(±0.00)	22.00(±0.00)
15 wt% K_3PO_4	Bottom	2.00	1.29(±0.06)	0.05(±0.01)	70.26(±3.09)	9.66(±1.72)	96.28(±0.48)	3.72(±0.48)
	Top	2.00	0.05(±0.02)	0.25(±0.01)	2.84(±0.93)	49.06(±2.63)	16.83(±3.90)	83.17(±3.90)
	Precipitate*	---	0.49(±0.04)	0.21(±0.00)	26.90(±2.15)	41.28(±0.90)	69.74(±2.15)	30.26(±2.15)

2. Effect of concentration of phase-forming compounds on TPP performance

2.1.Polymer-based Three Phase Partitioning

Table S4: Yields and purity of EPS-PS and EPS-PN fractionated by PEG-based TPP. Results reported as the average of two independent assays with their respective standard deviation. n/a = not applicable, * = not possible to accurately measure as observed volume of precipitate was most of the cases < 0.125 mL

System	Phase	Volume (mL)	EPS_{PS} (mg)	EPS_{PN} (mg)	Yield EPS_{PS} (%)	Yield EPS_{PN} (%)	Purity EPS_{PS} (%)	Purity EPS_{PN} (%)
32 wt% PEG1000	Initial	n/a	1.69(±0.00)	0.48(±0.00)	n/a	n/a	78.00(±0.00)	22.00(±0.00)
14 wt% $K_3C_6H_5O_7$	Bottom	1.20	0.93(±0.09)	0.01(±0.00)	54.72(±5.17)	2.80(±0.26)	98.57(±0.26)	1.43(±0.26)
	Top	3.40	0.25(±0.05)	0.12(±0.00)	14.92(±2.99)	25.26(±0.68)	67.41(±3.83)	32.59(±3.83)
	Precipitate*	---	0.51(±0.04)	0.34(±0.00)	30.37(±2.18)	71.95(±0.94)	59.90(±2.04)	40.10(±2.04)
28 wt% PEG1000	Initial	n/a	1.82(±0.00)	0.51(±0.00)	n/a	n/a	78.00(±0.00)	22.00(±0.00)
14 wt% $K_3C_6H_5O_7$	Bottom	0.80	1.07(±0.02)	0.02(±0.00)	59.07(±1.24)	3.87(±0.13)	98.18(±0.10)	1.82(±0.10)
	Top	3.80	0.24(±0.00)	0.10(±0.00)	13.20(±0.22)	18.61(±0.63)	71.55(±1.02)	28.45(±1.02)
	Precipitate*	---	0.50(±0.02)	0.40(±0.00)	27.72(±1.02)	77.52(±0.50)	55.90(±0.75)	44.10(±0.75)
20 wt% PEG1000	Initial	n/a	1.56(±0.00)	0.44(±0.00)	n/a	n/a	78.00(±0.00)	22.00(±0.00)
25 wt% $K_3C_6H_5O_7$	Bottom	2.50	0.88(±0.00)	0.04(±0.01)	56.02(±0.25)	8.12(±2.00)	96.08(±0.95)	3.92(±0.95)
	Top	2.00	0.19(±0.01)	0.09(±0.01)	11.99(±0.74)	20.44(±3.17)	67.56(±4.75)	32.44(±4.75)
	Precipitate*	---	0.50(±0.01)	0.31(±0.02)	31.99(±0.98)	71.45(±5.18)	61.36(±2.44)	38.64(±2.44)
20 wt% PEG1000	Initial	n/a	2.34(±0.14)	0.53(±0.00)	n/a	n/a	81.58(±0.91)	18.42(±0.91)
20 wt% $K_3C_6H_5O_7$	Bottom	2.30	1.87(±0.00)	0.08(±0.01)	80.07(±4.87)	16.01(±1.98)	95.68(±0.51)	4.32(±0.51)
	Top	2.20	0.20(±0.01)	0.16(±0.02)	8.69(±1.02)	29.98(±3.75)	56.25(±1.68)	43.75(±1.68)
	Precipitate*	---	0.27(±0.15)	0.29(±0.03)	11.24(±5.89)	54.01(±5.74)	46.33(±12.42)	53.67(±12.42)

2.2.Alcohol-based Three Phase Partitioning

Table S5: Yields and purity of EPS-PS and EPS-PN fractionated by EtOH-based TPP. Results reported as the average of two independent assays with their respective standard deviation. n/a = not applicable, * = not possible to accurately measure as observed volume of precipitate was most of the cases < 0.125 mL

System	Phase	Volume (mL)	EPS_{PS} (mg)	EPS_{PN} (mg)	Yield EPS_{PS} (%)	Yield EPS_{PN} (%)	Purity EPS_{PS} (%)	Purity EPS_{PN} (%)
34 wt% EtOH	Initial	n/a	1.95(±0.00)	0.55(±0.00)	n/a	n/a	78.00(±0.00)	22.00(±0.00)
16 wt% $K_3C_6H_5O_7$	Bottom	1.50	1.27(±0.05)	0.09(±0.01)	65.00(±2.40)	16.44(±1.01)	93.34(±0.15)	6.66(±0.15)
	Top	3.30	0.08(±0.00)	0.19(±0.01)	4.27(±0.15)	33.76(±1.70)	30.99(±1.82)	69.01(±1.82)
	Precipitate*	---	0.60(±0.05)	0.27(±0.00)	30.73(±2.55)	49.80(±0.70)	68.58(±2.09)	31.42(±2.09)
27 wt% EtOH	Initial	n/a	1.87(±0.00)	0.53(±0.00)	n/a	n/a	78.00(±0.00)	22.00(±0.00)
20 wt% $K_3C_6H_5O_7$	Bottom	1.75	1.36(±0.02)	0.07(±0.02)	72.39(±0.93)	13.21(±4.25)	95.11(±1.56)	4.89(±1.56)
	Top	2.00	0.10(±0.00)	0.10(±0.00)	5.42(±0.07)	18.20(±0.38)	51.36(±0.21)	48.64(±0.21)
	Precipitate*	---	0.42(±0.02)	0.36(±0.02)	22.19(±0.86)	68.59(±4.63)	53.43(±2.64)	46.57(±2.64)
18 wt% EtOH	Initial	n/a	1.62(±0.00)	0.46(±0.00)	n/a	n/a	78.00(±0.00)	22.00(±0.00)
30 wt% $K_3C_6H_5O_7$	Bottom	2.65	1.18(±0.01)	0.05(±0.02)	72.83(±0.71)	10.97(±4.08)	95.94(±1.42)	4.06(±1.42)
	Top	0.80	0.06(±0.00)	0.04(±0.00)	3.93(±0.05)	9.76(±0.23)	58.85(±0.28)	41.15(±0.28)
	Precipitate*	---	0.38(±0.01)	0.36(±0.02)	23.24(±0.66)	79.28(±3.84)	50.97(±0.51)	49.03(±0.51)
23 wt% EtOH	Initial	n/a	1.62(±0.00)	0.46(±0.00)	n/a	n/a	78.00(±0.00)	22.00(±0.00)
25 wt% $K_3C_6H_5O_7$	Bottom	2.25	1.33(±0.10)	0.05(±0.00)	81.89(±5.85)	10.21(±1.09)	96.58(±0.59)	3.42(±0.59)
	Top	1.40	0.07(±0.01)	0.06(±0.00)	4.58(±0.35)	13.67(±0.48)	54.28(±1.03)	45.72(±1.03)
	Precipitate*	---	0.22(±0.10)	0.35(±0.00)	13.53(±6.20)	76.13(±0.61)	37.69(±11.23)	62.31(±11.23)
